# Hybrid closed‐loop systems in UK type 1 diabetes care: National survey of healthcare professional awareness, confidence, and training needs

**DOI:** 10.1111/dom.70027

**Published:** 2025-08-22

**Authors:** Ananthi Anandhakrishnan, Alexandros L. Liarakos, Ketan Dhatariya, Geraldine Gallen, Pratik Choudhary, Alistair Lumb, Emma G. Wilmot, Sufyan Hussain

**Affiliations:** ^1^ Department of Diabetes and Endocrinology Guy's and St Thomas' NHS Foundation Trust London UK; ^2^ Department of Diabetes, School of Cardiovascular, Metabolic Medicine and Sciences King's College London London UK; ^3^ Department Of Diabetes And Endocrinology University Hospitals of Derby and Burton NHS Foundation Trust Derby UK; ^4^ Faculty Of Medicine And Health Sciences University of Nottingham Nottingham UK; ^5^ Elsie Bertram Diabetes Centre Norfolk and Norwich University Hospitals NHS Foundation Trust Norwich UK; ^6^ Norwich Medical School University of East Anglia Norfolk UK; ^7^ Department of Diabetes King's College Hospital NHS Foundation Trust London UK; ^8^ University Hospitals of Leicester NHS Foundation Trust Leicester Diabetes Centre Leicester UK; ^9^ Oxford Centre for Diabetes Endocrinology and Metabolism Oxford University Hospitals NHS Trust Oxford UK; ^10^ National Institute for Health and Care Research Oxford Biomedical Research Centre Oxford UK; ^11^ Institute of Diabetes, Endocrinology and Obesity King's Health Partners London UK

**Keywords:** awareness, confidence, healthcare professional, hybrid closed loop system, survey, training needs, type 1 diabetes

## Abstract

**Aims:**

Hybrid closed‐loop (HCL) systems significantly improve glycaemia and have become the standard of care for type 1 diabetes (T1D), leading to the recent widescale implementation programme in England and Wales. Limited data exist regarding UK healthcare professionals' (HCPs) confidence and experience with commercially available HCL systems. This survey aimed to evaluate UK HCPs' awareness, confidence, and training needs concerning commercial HCL systems.

**Materials and Methods:**

A national survey, developed in collaboration with the Association of British Clinical Diabetologists' Diabetes Technology Network (ABCD‐DTN‐UK), was distributed to adult and paediatric diabetes care teams. Data were collected between July and November 2024 and analysed descriptively, stratified by professional roles and care settings.

**Results:**

Responses from 637 HCPs (42.4% diabetes specialist nurses, 24.5% endocrinologists, 16.6% dieticians, 12.7% endocrinology residents) across 135 healthcare organisations in the UK revealed high overall awareness regarding HCL initiation and safety. Awareness varied significantly by role, with endocrinologists and diabetes nurse specialists reporting greater familiarity than endocrinology residents (*p* < 0.001). Confidence in system‐specific use varied, with notably lower confidence in pregnancy‐specific CamAPSFx‐based systems. 41% of respondents involved in pregnancy care reported no confidence in advising on these systems, with 7% reporting no access to any CamAPSFx‐based system. A majority (73.3%) expressed interest in additional training, favouring remotely accessible modules (52.3%).

**Conclusions:**

This survey highlights encouraging overall confidence and awareness of commercial HCL systems among UK HCPs, reflecting successful recent educational initiatives. However, significant gaps, particularly concerning pregnancy‐licensed systems and variable exposure to specific HCL systems, identify clear training needs. Addressing these will be crucial in ensuring equitable and effective HCL implementation of systems licensed for use across the lifespan of an individual with T1D and across diverse clinical settings.

## 1 INTRODUCTION

Despite advancements in the management of type 1 diabetes (T1D), the daily management burden is significant. It is unsurprising therefore, that for the majority of people living with T1D (pwT1D), glycaemia remains above recommended targets. Data from the 2021–2022 National Diabetes Audit in England and Wales highlights that less than one third of adults with T1D meet conservative treatment targets for their condition (haemoglobin A1c; HbA1c ≤7.5%).^1^ Similarly, in the United States, data from the 2018 Diabetes Exchange registry reports that only 21% of adults with T1D attain HbA1c targets of below 7%.^2^ Global data further reinforces these experiences, with observational data from the SAGE study reporting that only 24.3% of adults with T1D from a multinational cohort attained HbA1c targets of below 7%.^3^

Hybrid closed‐loop (HCL) systems are a form of automated insulin delivery that use continuous glucose monitoring (CGM) data to direct algorithm‐based insulin delivery via an insulin pump.^4^ They represent a major advancement in diabetes management, and are now considered the standard of care for people with T1D. Currently available commercial HCL systems have consistently demonstrated improvements in glycaemia in clinical trial^5^, ^6^, ^7^, ^8^, ^9^, ^10^, ^11^, ^12^, ^13^, ^14^ and real‐world^15^, ^16^, ^17^, ^18^, ^19^, ^20^, ^21^ settings, with associated reductions in diabetes burden, and improved wellbeing.^22^ Their use has been assessed by a comprehensive landmark health economic technology appraisal (TA) by the National Institute for Health and Care Excellence (NICE) in England.^23^ This NICE TA was accompanied by a 5‐year implementation programme, with planned rapid uptake of HCL by between 60 and 75 000 people over the next 5 years.^24^

To deliver this national programme, there is growing recognition of the need for HCPs to be adequately equipped with the necessary skills and experience to enable the full potential of HCL systems in pwT1D.^25^ The Diabetes Technology Network United Kingdom (DTN‐UK) is a voluntary national organisation focused on supporting access to diabetes technologies, and has undertaken a comprehensive programme of education and resource development. These initiatives include the publication of a best practice guide offering expert consensus advice for (HCPs) on commercially available HCL systems in the UK^25^; the development of widely accessed patient‐facing educational resources; the continuing professional development (CPD) approved Glooko Academy programme^26^; face‐to‐face and online training courses; and the provision of ongoing online CPD programmes.^25^, ^26^, ^27^ However, the effectiveness of these initiatives and the current competency and educational requirements among HCPs remain unknown.

The aim of the present study was to explore HCP confidence and awareness regarding HCL implementation in routine clinical care. HCP experiences were assessed against key areas outlined in the DTN HCL best practice guide, which relate to the use of commercial HCL systems in T1D care.^25^ By identifying common challenges and gaps in confidence or awareness, findings will inform training needs and requirements for future educational initiatives that may support more effective implementation of HCL technologies in diverse healthcare settings.

## 2 METHODS

### 2.1 Survey design

A survey was developed by a group of diabetes specialists designed to evaluate HCPs awareness, confidence, and exposure to commercially available HCL systems. The survey was directed towards HCPs providing T1D care in the UK and aimed to define training needs. The survey was approved by the Association of British Diabetologists–Diabetes Technology Network UK (ABCD‐DTN‐UK) committee. Ethical approval was obtained from King's College London Research Ethics Committee (MRA 20/21‐24 910). Participation in the survey was voluntary; as such, completion was deemed consent to participate.

### 2.2 Questionnaire content

A questionnaire (Supplemental information) was designed, comprising five domains that aimed to assess confidence and awareness in the following areas: (i) HCL initiation and patient education, (ii) data interpretation and troubleshooting technical issues, (iii) safety considerations, (iv) use of HCL in pregnancy care, perioperatively, as an inpatient and during exercise, and (v) current training and future needs regarding commercially available HCL systems. Domains were adapted from key areas related to the use of commercial HCL systems in T1D care as outlined in the DTN best practice guidelines.^25^ The survey also included questions on the demographics, professional roles, and work environment of participating HCPs, as well as their current levels of exposure to commercially available HCL systems. A brief section at the end of the survey assessed the extent of perceived inequities in the provision of HCL systems, the effectiveness of mitigation strategies, and potential widescale HCL implementation challenges.

A combination of nominal scales, ordinal scales (respondents of the survey were asked to rate confidence, awareness and effectiveness on a 7‐point Likert scale: 1 = not at all confident/aware/effective; 4 = moderately confident/aware/effective; 7 = completely confident/aware/effective), and free text boxes were used to gather qualitative and quantitative data.

### 2.3 Survey distribution

The survey was distributed via the ABCD and ABCD‐DTN‐UK. The survey was disseminated via email to ABCD‐DTN UK members (1995 healthcare professionals [HCPs]) and ABCD members (717 HCPs). The Association of Children's Diabetes Clinicians (ACDC), the Young Diabetologists and Endocrinologists' Forum (YDEF), the Diabetes Specialist Nurse (DSN) Forum, and the King's Health Partners Diabetes Technology Course assisted in publicising the study.

Data were collected using an electronic data capture tool hosted on the ABCD‐DTN‐UK website. The survey took place from 26 July 2024 to 4 November 2024.

### 2.4 Inclusion and exclusion criterion

HCPs involved in the care of individuals with T1D practicing in the UK were included in the survey. Non‐HCPs and those not disclosing their profession were excluded.

### 2.5 Data analysis

Anonymous data collected as part of the national survey were used for analysis. Data were summarised using descriptive statistics and reported as mean ± standard deviation (SD) or median with interquartile range [IQR], depending on the distribution. Normality was assessed using the Kolmogorov–Smirnov test. Comparisons between two independent groups were conducted using the independent *t*‐test for normally distributed continuous variables and the Mann–Whitney *U* test for non‐normally distributed or ordinal data. The chi‐squared test was used to assess differences in proportions across two or more independent groups for categorical variables. A *p*‐value of <0.05 was considered statistically significant; all tests were two‐sided. Statistical analyses were conducted using SPSS software, version 29.0.2.0 (IBM Corp.).

### 2.6 Terminology

The term ‘endocrinologist’ is used throughout this text to refer broadly to medical specialists involved in the care of people with diabetes. This includes diabetologists, specialist diabetes physicians, and consultants in diabetes and endocrinology.

The term ‘endocrinology residents’ is used throughout this text to refer broadly to doctors specialising in diabetes and endocrinology (speciality trainee year 3–7, or equivalent higher‐level residents or fellows).

The terms NHS Trusts, NHS Boards, and Health and Social Care Trusts refer to the administrative bodies responsible for delivering specialist hospital and community health services in England, Scotland, and Wales, and Northern Ireland, respectively. Each NHS Trust, NHS Board, or Health and Social Care Trust may encompass multiple centres. The term general practice is used to denote primary care services, while private practice refers to clinics that operate independently of public funding within the UK.

## 3 RESULTS

### 3.1 Respondents

Six‐hundred and forty‐two respondents participated in the survey. Responses from five participants were excluded from analysis: 1 dispenser; 1 administrator; 1 youth worker; and 2 individuals who did not indicate a professional role (Figure S1), leaving a total cohort of 637 HCPs from 135 healthcare organisations across the UK. Table 1 presents the demographics, professional roles and workplaces of surveyed participants, alongside the age groups of pwT1D whose care they are involved with.

**TABLE 1:** Demographics, professional role and workplaces of respondents, alongside age groups of people with type 1 diabetes whose care respondents are involved with.

Category	Number	Percentage (%)
Age of respondents		
18–25	4	0.6
26–35	102	16
36–45	204	32
46–55	216	33.9
56–70	106	16.6
>70	2	0.3
No data reported	3	0.5
Gender of respondents		
Female	505	79.3
Male	121	19
Prefer not to say	7	1.1
No data reported	4	0.6
Profession of respondents		
Diabetes specialist nurses	270	42.4
266 Diabetes nurses		
3 diabetes nurse endocrinologists		
1 diabetes matron		
Diabetes dieticians	106	16.6
Endocrinologists	156	24.5
Endocrinology residents	81	12.7
Other	24	3.8
9 psychologists		
5 midwives		
3 pharmacists		
1 podiatrist		
1 practice nurse		
1 diabetes research nurse		
1 clinical educator		
1 physicians associate		
1 obstetrician		
1 advanced clinical practitioner		
Age group of populations managed by respondents		
Adults and young adults	224	35.2
Paediatrics (mostly under 19)	216	33.9
Adults	161	25.3
Young adults (18–25)	13	2
Young adults and paediatrics	13	2
Adults and paediatrics	10	1.6
No data reported	0	0
Place of work		
Teaching hospital	346	54.3
District general hospital	250	39.2
Community Diabetes service	30	4.7
GP surgery	5	0.8
Private practice	5	0.8
No data reported	1	0.2

The majority of respondents, 93.5%, worked in secondary (specialist) care services (54.3% university teaching hospitals, 39.2% district general hospitals). Among respondent, 42.4% (270/637) were diabetes specialist nurses (DSNs); 24.5% (156/637) were endocrinologists; 16.6% (106/637) were diabetes dieticians; 12.7% (81/637) were endocrinology residents, with other HCPs accounting for the remaining 3.8% (24/637).

The majority of respondents worked in adult services, either alone (25.3%) or alongside young‐adult (35.2%) or paediatric services (2%). A total of 33.9% worked in paediatric services alone, 68.8% (438/637) worked in trusts/clinics with specific clinics for pwT1D using insulin pump therapy, including HCL systems, and 62.6% (399/637) were involved in the inpatient care of individuals with T1D using HCL systems. The median number of weekly diabetes outpatient clinics performed by respondents was 2 [1, 4] with 9.2% of the cohort involved in more than five clinics a week (Figure S2A). Forty‐one percent (262/637) of the cohort reported encounters with more than 6 pwT1D in the outpatient setting weekly (Figure S2B).

Places of work were grouped according to NHS Trust (England), NHS Board (Scotland and Wales), or Social Care Trust (Northern Ireland) to include specialist services within each nation of the UK respectively. Primary care and private practices were categorised separately (see Method; Terminology). Of the 135 sites included, 116 were NHS Trusts (86%), 7 were Health Boards in Scotland (5.1%), 6 were Health Boards in Wales (4%), and 2 were Social Care Trusts (1.5%). Individuals from two private practices and two general practices all located in England also contributed to the survey.

Respondents from the 116 NHS Trusts in England accounted for 411 of the 455 respondents disclosing their region of work (90.3%). Services within the Republic of Ireland, Wales, and Scotland contributed a combined total of 40 respondents (8.8%). One‐hundred and eighty‐two respondents (28.6%) did not disclose their region of work. The highest number of respondents from a single centre was 16, with a median number of respondents from each organisation of 2 [1, 5]. Table S1 provides a breakdown of the number of respondents from each NHS Trust, NHS Board, Social Care Trust, Private Practice, and GP Practice.

### 3.2 HCL starts and patient education

Four‐hundred and thirty‐one respondents (67.7%) were involved in HCL starts within their place of work. Across the total cohort, awareness of the procedures for initiating HCL was rated highly, with a median score of 7 [5, 7] on a 1–7 Likert scale. Awareness varied by place of work and professional role. Respondents from district general hospitals reported greater awareness (7 [6, 7]) when compared with their peers in teaching hospitals (6.5 [5, 7]) (*p* = 0.016). Endocrinologists were more aware (7 [6, 7]) when compared with endocrinology residents (5 [3, 6]) (*p* < 0.001) with DSNs (7 [6, 7]) significantly more aware than both endocrinologists (*p* < 0.001) and endocrinology residents (p < 0.001).

**FIGURE 1:**
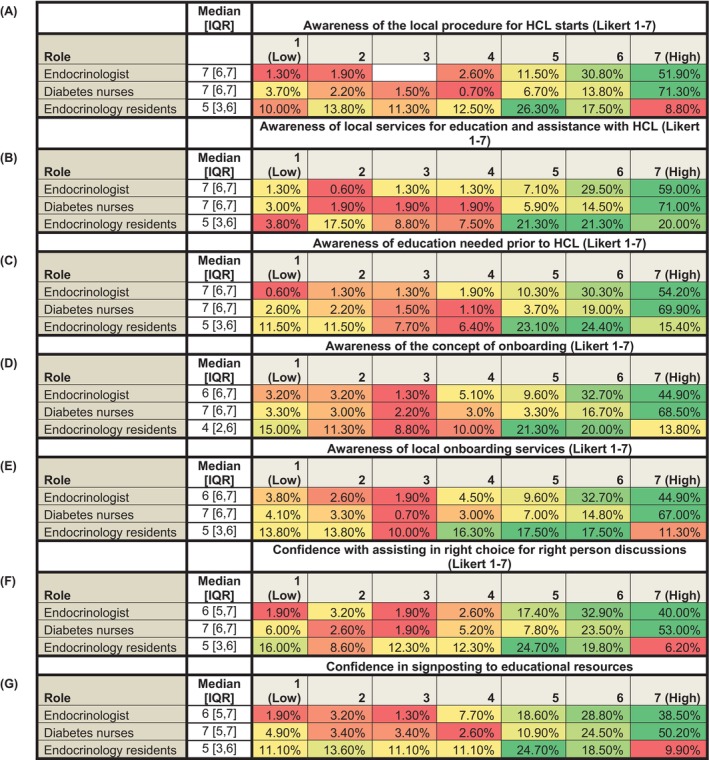
Heatmaps depicting awareness and confidence related to hybrid closed‐loop (HCL) starts and patient education, stratified by professional role. Respondents were stratified based on their reported professional role. Confidence was rated on a 7‐point Likert scale (1 = Low, 7 = High), and colour intensity reflects the proportion of responses within each group, from red (lower proportion) to green (higher proportion). Endocrinology residents reported reduced awareness of: A, the local procedure for HCL starts; B, services available locally for education people with type 1 diabetes about HCL; C, the education needed prior to HCL; D, the concept of onboarding; E, the local onboarding services, and confidence with; F, assisting a person with type 1 diabetes (T1D) in making a decision about which HCL system would be right for them, and in; G, signposting a person with T1D considering HCL towards educational resources when compared with Diabetes nurses and Endocrinologists (*p* < 0.001 for all comparisons between endocrinology residents with Diabetes nurses, and endocrinology residents and endocrinologists). Diabetes nurse reported greater awareness when compared with endocrinologists for: C, the education needed prior to HCL (*p* = 0.003); D, the concept onboarding (*p* < 0.001); and E, the local onboarding services (*p* < 0.001). Median [interquartile range (IQR)] values are provided for each professional role within each domain.

Across the total cohort, awareness of available services to support and educate pwT1D prior to commencing HCL therapy was rated highly (7 [6, 7]), as was familiarity with the onboarding process for HCL systems (7 [5, 7]) and the resources available for this within a given clinic or trust (6 [5, 7]). Confidence in supporting a person with T1D in choosing the right HCL system for them was also rated highly (6 [5, 7]) as was confidence in signposting individuals to relevant online educational and training platforms (6 [5, 7]). Place of work did not influence confidence or awareness of HCL starts and patient education, which was found to vary with professional role (Figure 1).

### 3.3 Currently available systems

**FIGURE 2:**
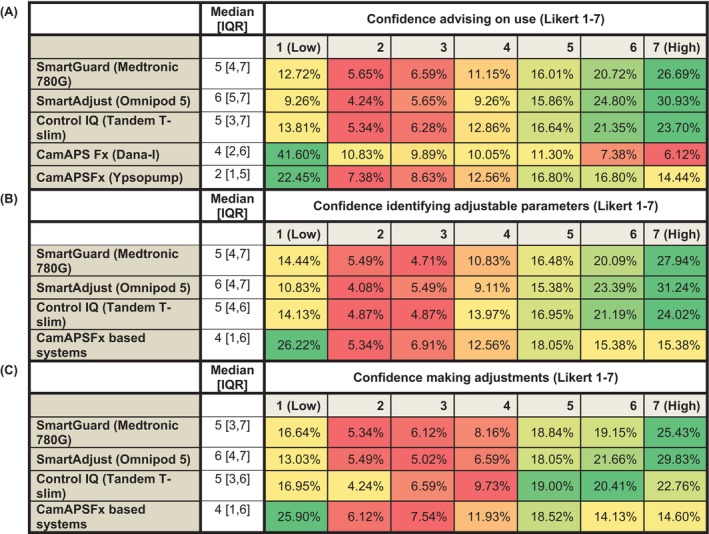
Heatmaps representing healthcare professionals' confidence across three domains related to hybrid closed‐loop (HCL) systems: A, advising on use; B, identifying adjustable parameters, and; C, making adjustments. Responses are based on a 7‐point Likert scale from 1 (Low confidence) to 7 (High confidence). Colours range from red (lower proportions within each row) to green (higher proportions), indicating relative frequency of responses for each HCL system: SmartGuard (Medtronic 780G), SmartAdjust (Omnipod 5), Control IQ (Tandem T‐slim), and CamAPSFx (Dana‐I and Ypsopump). CamAPSFx data are grouped separately where applicable. Median [interquartile range (IQR)] values are provided for each system within each domain.

Confidence in advising pwT1D on the use of current commercially available HCL systems varied with system (Figure 2). Confidence across the total cohort was high for the Control IQ via Tandem T‐slim (CIQ/T‐slim) (5 [3, 6]), Medtronic 780G HCL system using SmartGuard technology (780G) (5 [4, 7]) and Omnipod® 5 HCL system with SmartAdjust technology (OP5) (6 [4, 7]). Lower confidence was reported for the CamAPSFX system when used with both the Ypsopump as myLife Loop (CamAPSFx/Ypso) (4 [2, 6]) and Dana‐I (CamAPSFx/Dana) (2 [1, 5]), with 22.3% (143/637) and 41.5% (265/637) of the total cohort, respectively, reporting to be not at all confident (Likert = 1).

The majority of respondents (54.9% [350/637]) indicated they were very confident (Likert score ≥6) in their awareness of how commercially available HCL systems determine insulin delivery, and were similarly confident (64.5% [413/637] with Likert ≥6) in their awareness of what glucose target ranges can be specified within each system. Awareness of how each system modulates basal insulin delivery and administers automated correction boluses in response to hyperglycaemia was also rated highly (6 [6, 7]) as was awareness of which parameters were adjustable within each system and how these influenced glycaemia (6 [5, 7]).

Confidence in identifying and adjusting modifiable parameters varied between system (Figure 2). Median confidence scores for both identifying and adjusting modifiable parameters for CamAPSFX‐based systems was (4 [1, 6]), with 26.2% (167/637) and 25.9% (165/637) of respondents reporting to be ‘not at all confident’ (Likert = 1) with identifying and adjusting modifiable parameters within CamAPSFX‐based systems respectively.

### 3.4 HCL use in antenatal care

Across the total cohort, confidence in advising on the use of HCL systems during pregnancy was generally low, with a median Likert score of 3 [1, 5]. Of the 637 respondents, 39.6% (*n* = 252) reported involvement in the care of pwT1D using HCL systems during pregnancy. This group included 100 diabetes specialist nurses (DSNs), 52 endocrinology residents, 46 endocrinologists, 45 diabetes dietitians, and 9 respondents from other professional roles. Among them, 97.6% (246/252) reported being either aware (92.2%; 227/246) or somewhat aware (7.8%; 19/246) of which HCL systems are licensed for use during pregnancy. Confidence in advising on HCL use in pregnancy was significantly higher in this group, with a median Likert score of 6 [4, 6], compared to those not involved in such care (48.8%; 311/637), *p* < 0.001. In this group median confidence was 1 [1,3] and 62.5% (*n* = 193/311) reported being not at all confident in advising on HCL use in pregnancy (Likert = 1). Among those reporting no involvement in the care of pwT1D using HCL systems during pregnancy, 69.5% (216/311) reported being either aware (72.2%; 156/216) somewhat aware (27.8%; 60/216) of which systems are licensed for use in pregnancy, Awareness was significantly associated with involvement in antenatal diabetes care (χ^2^ = 105, *p* < 0.001). Respondents who selected ‘not applicable’ (*n* = 73) or did not respond to the question (*n* = 1) were excluded from this comparison. DSNs and endocrinologists were similarly confident in advising on HCL use in pregnancy (*p* = 0.150), with both groups more confident than endocrinology trainees (*p* = 0.001 and *p* = 0.045 for comparisons between DSNs and endocrinology trainees, and endocrinologists and endocrinology trainees respectively).

**FIGURE 3:**
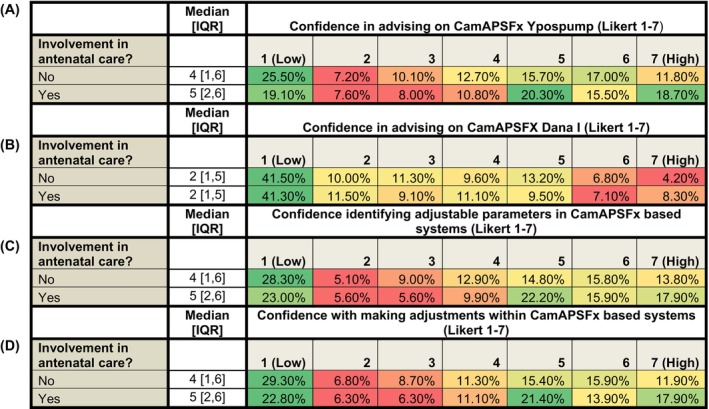
Heatmaps illustrating healthcare professionals' confidence across four domains related to pregnancy‐specific hybrid closed‐loop (HCL) systems (CamAPSFx): A, advising on use via Ypsopump; B, advising on use via Dana‐I; C, identifying adjustable parameters within CamAPSFx based systems and; D, making adjustments within CamAPSFx based systems. Respondents were stratified based on their reported involvement in antenatal diabetes care for people with type 1 diabetes (pwT1D). Confidence was rated on a 7‐point Likert scale (1 = Low, 7 = High), and colour intensity reflects the proportion of responses within each group, from red (lower proportion) to green (higher proportion). A total of 637 respondents participated in the survey; 252 (39.6%) reported involvement in antenatal care, while 311 (48.8%) did not. Those involved in antenatal care reported significantly higher confidence in advising on the use of the CamAPSFX Ypsopump (*p* = 0.016), identifying adjustable parameters within CamAPSFx based systems (*p* = 0.039), and in making these adjustments (*p* = 0.012). Confidence remained low across both groups when advising on use via the Dana‐i pump, with over 40% in each cohort selecting “not at all confident” (Likert = 1), *p* = 0.639. Median [interquartile range (IQR)] values are provided for each cohort within each domain.

Among individuals involved in the care of pwT1D using HCL systems during pregnancy, significantly higher confidence was reported compared to those not involved in such care. The group demonstrated greater confidence in identifying which parameters are adjustable within CamAPSFX‐based systems (*p* = 0.039), in making those adjustments (*p* = 0.012), and in advising on the use of the CamAPSFx/Ypso (*p* = 0.016). Confidence in advising on the use of the CamAPSFx/Dana did not differ significantly between the groups (*p* = 0.632), with 41.3% (104/252) of those involved in pregnancy‐related HCL use reporting being not at all confident (Likert score = 1) (Figure 3).

Confidence in the use of CamAPSFx‐based systems was significantly higher among respondents with access to these systems within their place of work. Among the total cohort, 74.1% (472/637) reported having access to CamAPSFX/Ypso. These respondents reported significantly higher confidence in advising on the use of CamAPSFX/Ypso (5 [4, 6]), compared to those without access (12.9%; 82/637), who had a median score of 1 [1, 2.75] (*p* < 0.001). 62.2% (51/82) of respondents without access reported being not at all confident (Likert = 1). Similarly, respondents with access to CamAPSFX/Ypso also demonstrated significantly greater confidence in advising on the use of CamAPSFX/Dana (*p* < 0.001), in identifying which parameters are adjustable within CamAPSFX‐based systems (*p* < 0.001), in making these adjustments (*p* < 0.001), and in advising on the management of glycaemia in pregnancy in people with T1D using HCL systems (*p* = 0.020).

Among the total cohort, 34% (217/637) reported having access to CamAPSFX/Dana. These respondents reported significantly higher confidence in advising on the use of CamAPSFX/Dana (5 [2, 6]) when compared to those without access (40%; 257/637), who had a median score of 1 [1, 3] (*p* < 0.001). 57.2% (147/257) of respondents without access reported being not at all confident (Likert = 1). Similarly, respondents with access to CamAPSFX/Dana also demonstrated significantly greater confidence in advising on the use of CamAPSFX/Ypso (*p* = 0.018), in identifying which parameters are adjustable within CamAPSFX‐based systems (*p* < 0.001), in making these adjustments (*p* < 0.001), and in advising on the management of glycaemia in pregnancy in people with T1D using HCL systems (*p* = 0.012).

Of the 252 individuals involved with the use of HCL during antenatal care, 10.3% (26/252) reported no access to the CamAPSFx/Ypso, and 37.3% (94/252) reported no access to CamAPSFx/Dana systems within their department. 7% (17/252) reported no access to either system. In addition, 9.9% (25/252) and 22.6% (57/252) were unsure whether access to the CamAPSFx/Ypso or CamAPSFx/Dana systems, respectively, was offered in their department.

### 3.5 Safety considerations

Awareness of safety considerations surrounding the use of HCL was rated highly across the group, with over 60% of the cohort rating themselves very or completely aware (Likert ≥6) of each of the following circumstances: the circumstances that should prompt consideration of cannula failure (6 [5, 7]); the management of sick day rules and unexplained hyperglycaemia (6 [5, 7]); hypoglycaemia management when on HCL (6 [5, 7]); when to advise a pwT1D to switch from HCL to manual mode (6 [5, 7]) and when to switch to multiple daily dose insulin (MDI) (6 [5, 7]). Spearman's correlation analyses revealed significant relationships between awareness in all safety considerations detailed above (Table S3). Knowledge of sick day rules showed strong correlations with all other areas, particularly with hypoglycaemia management (R = 0.868, *p* < 0.001) and the decision to switch to MDI (R = 0.855, p < 0.001). The strongest correlation observed was between understanding when to use manual mode and when to transition to MDI (R = 0.924, p < 0.001). There were differences in the levels of awareness reported when the cohort was grouped according to role, with endocrinologists and DSNs' reporting greater awareness of safety considerations when compared with endocrinology residents (Table S4).

Free text responses were provided to review what strategies respondents were aware of to avoid problematic hyperglycaemia. Common themes included: reference to ketone monitoring and sick day rules; management guidelines provided by the DTN‐UK^25^, ^28^ and structured education programmes for T1D (dose adjustment for flexible eating; DAFNE)^29^, ^30^; alarms on HCL systems; pod or cannula changes and rotation of sites; reverting to MDI; and adequate provision of education from HCPs to pwT1D.

### 3.6 Special circumstances

Confidence in managing or providing advice on HCL use in pwT1D was rated highly for use during exercise (5 [4, 6]) and for inpatient care (5 [3, 6]) and rated fair for perioperative use (4 [2, 6]). 53.8% (343/637) of the respondents reported involvement in the inpatient care of pwT1D undergoing surgery. This group reported significantly higher confidence in the perioperative management of pwT1D using HCL (5 [4, 6]) than those not (145/637) (3 [1, 5]) (*p* < 0.001).

### 3.7 Current training and future needs

Just under three‐quarters (73.3%, 467/637) of respondents stated that they would like further training on HCL therapies. In this cohort, remotely accessible modules were reported as the top preference for over half (52.3%, 244/467) and the second choice in 14.3% (67/467). Face‐to‐face locally delivered teaching was the second most popular first choice, the top choice in 26.8% (128/467).

### 3.8 Access and implementation

When respondents were asked to rate the inequities in the provision of HCL in current service on a scale of 1–5 (none to extreme), 11.3% (72/637) reported no inequalities whilst 26% (166/637) reported significant or extreme inequalities (Likert ≥4). The median response was 2 [2, 4] with the majority of individuals (62.3% [397/637]) reporting the presence of some Likert = 2 (40.3% [257/637]) or considerable Likert = 3 (2% [140/637]) inequalities. Strategies to overcome these included tailored education for those with reduced digital literacy; easy to read written materials; consultations assisted by translation and interpretation services; educational resources provided in common non‐English languages; alterations in product design for those with visual impairment; and alterations in product design for those with dexterity issues. These were all rated highly in their perceived efficacy in targeting current inequities (47.1% [300/637] to 54.5% [347/637] of respondents rating a Likert ≥6 [very or extremely effective] for each domain).

Free text responses were provided to understand issues that may impact the implementation of HCL systems within services. A content analysis of the responses identified several key issues detailed in Table 2. Staffing shortages, alongside limited trainer availability and administrative support, were frequently cited as major barriers. Funding constraints and complexities significantly hindered service capacity and caused regional disparities in access to devices and technologies. Logistical challenges such as limited clinic space and high patient volumes further limited the ability to onboard and support patients effectively.

**TABLE 2:** Content analysis from free‐text responses from participants regarding issues that may impact HCL implementation.

Category	Key points	Illustrative quotes
Staffing and workforce	Lack of specialists adequately trained in HCL implementation Lack of trainers Lack of administrative staff support	*‘Lack of training for specialty doctors, …low staffing of DSNs and overall team to meet the demand of local population’* *‘More time is needed to start HCL and more frequent contacts needed initially – lack of staffing to handle this increased workload.’* ‘we are a hub service but still struggle with balancing staffing with demand’
Funding and financial	Funding delays, constraints and uncertainties Postcode lottery with regards funding allocation	*‘Differing commissioning policies …and financial restrictions being imposed on HCL onboarding’* *‘…(we have) really battled with those holding the purse strings not understanding what they are stopping us from providing…’* *‘finance issues around seeking reimbursement …have results in high admin workload for the department.’* *‘max of 2pts/month due to limited finances’*
Implementation logistics	Lack of adequate clinic capacity Long wait times to onboarding Insufficient workforce to patient ratio Insufficient clinic spaces;	*‘Lack of space to do group starts…’* *‘…lack of provision of clinics for it’* *‘Lack of adequate capacity in clinic leading to significant backlog …reduced ability to identify eligible patients and offer appropriate support and training’* ‘…(need) clinic space for outreach to people who are unable to travel’
Patient and equity factors	Language barriers Reluctance to take up technology Cultural issues Disparities in health and technology literacy Deprivation	*‘Language and culture understanding’* *‘…health inequality is our biggest barrier’* *‘High deprivation—not all families having smart technology/connectivity’* *‘…low educational level of parents/inability to understand the advice.’* *‘Reluctance of children and young people to want to use HCL.’* *‘HCL companies could do more to provide either a phone or dedicated device to facilitate HCL.’*
Technology challenges	Device suitability versus availability Needing Wifi connection within clinics to allow onboarding and data download Needing compatible mobile phones	*‘Locally we are recognising some risks associated with some systems for individual patients’* *‘Mobile phone and wifi signal in hospital clinic, phones for those with digital poverty…’* *‘we have a local charity which supplies cleaned up used phones but if they are waiting for donations then it can delay getting patients onto the pump’*
Training and education	Need for national competencies Tailored patient and carer education needs and having the time and capacity to deliver this Tailored training and education for those with language or sensory needs	*‘Need for nationally produced clinical guidance for patients and for in‐patient teams.’* *‘We are giving HCL to anybody, so those families where literacy/language is an issue we are needing to help with this…lots more education sessions for them to understand the system. More help guides in different languages would be helpful.’*
Other	Some sites reporting no issues regards resource availability	*‘Majority already on HCL’* *‘We currently have 70% of our caseload on HCL and therefore don't find we have any issues apart from trying to get the pump‐resistant patients to contemplate it!’*

Patient‐related factors, including included technology aversion, varied health and digital literacy and socioeconomic deprivation were key factors influencing HCL implementation; one participant reported ‘health inequality is our biggest barrier—we have many patients who cannot afford a mobile device to run the tech on or if they can afford the device they are unable to use it safely’. This, in addition to the accessibility limitations of current systems in those for whom English is a second language raised concerns about equitable access. Additionally, respondents highlighted the need for nationally standardised training, clear clinical guidance, and enhanced educational resources to support both staff and patients. Despite these challenges, some services reported successful implementation with high uptake of HCL technology.

## 4 DISCUSSION

This survey, conducted during the first year of the NICE TA HCL roll out, provides important insights into UK HCPs experiences with commercially available HCL systems in routine T1D care. Overall, respondents reported high confidence and awareness in supporting patient selection, onboarding processes, and optimisation of clinical outcomes. However, notable gaps were identified, particularly concerning pregnancy‐related care and equitable training opportunities.

### 4.1 Supporting safe initiation and sustained use of HCL therapy: Onboarding, patient education, and safety considerations

As access to HCL systems increases, pwT1D should have the opportunity to select the system that best aligns with their needs.^23^ Informative discussions on system suitability during selection and effective troubleshooting following system start are only possible if HCPs are familiar with available systems. The ability to hold such discussions aligns with the minimum standards defined by the ABCD‐DTN‐UK standards of care for management of adults with T1D.^31^ It is reassuring, therefore, that across the cohort, there was a high level of reported awareness regarding local and wider services available for patient education and confidence in assisting individuals in selecting a suitable HCL system. It is worth noting that responses were limited by quantitative grading. Incorporating free‐text responses could offer a more detailed qualitative exploration of the factors HCPs consider important when supporting system choice.

Hypoglycaemia and unexplained hyperglycaemia management are identified as key considerations for using HCL systems in the DTN best practice guidelines.^25^ High awareness in the management of these scenarios is likely a reflector of access to existing national guidelines^25^, ^28^, ^29^ and educational resources.^32^, ^33^ Positive correlations between awareness of safety considerations suggest that those HCPs reporting high awareness in one aspect of management tend to feel similarly aware in others. However, the converse is also true: those who rate their awareness as low in one domain are likely to feel similarly unaware in related areas. These observed relationships suggest that perceived gaps in knowledge may reflect a broader lack of experience or preparedness with HCL system delivery. As such, it is of note that endocrinology residents as a whole reported reduced awareness surrounding HCL starts and patient education and safety considerations when compared with endocrinologists and DSNs. This may reflect the fact that pump services are often consultant led, and there are often limited opportunities for endocrinology residents to see patients on HCL independently. Findings highlight the interconnected nature of the principles surrounding safe HCL implementation and reinforce the need for sufficient protected time for training and hands‐on experience to adequately educate a specialist workforce to enable the safe and effective implementation of HCL systems.

### 4.2 Currently available systems

Confidence across the total cohort was reported as high for the CIQ/T‐slim, 780G and OP5 HCL systems. Whilst confidence in the use of CamAPSFx based systems was rated as low across the cohort, ratings were notably higher among respondents involved in antenatal T1D care. Although five HCL systems are currently commercially available in the UK and are intended to support the widescale implementation programme in England,^23^ the recent DTN statement recommends the Medtrum system should not be offered to individuals with T1D ‘until published evidence of safety and efficacy is provided by the company’.^34^ As such, experience with this system was not addressed as part of this survey.

### 4.3 Pregnancy‐specific HCL systems

Currently, only CamAPSFx‐based HCL systems are licensed for, and have evidence to support use in, pregnancy.^35^ The recent inclusion of improving access to pregnancy‐specific HCL systems into the ‘Saving Babies’ Lives Care Bundle’, in line with NICE TA recommendations, highlights the key role of HCL in maternal and foetal outcomes in T1D care.^36^ Despite this, access remains limited, with 10.3% and 37.3% of individuals involved in antenatal care reporting no access to the CamAPSFX/Ypso and CamAPSFX/Dana systems respectively, with 7% reporting no access to either system. These findings highlight a key gap in the equitable provision of evidence‐based advanced diabetes technologies in pregnancy.

Findings also support the need to incorporate guidance on pregnancy‐specific HCL system use into best practice guidelines for diabetes technology in pregnancy,^37^ currently under development. Additionally, these results highlight the need for further educational resources tailored to the Dana‐I pump. However, the reported low confidence among healthcare providers may reflect limited access to, and consequently limited experience with, the device; availability of the CamAPSFX/Dana system was reported by only 34% of the cohort.

### 4.4 Current training and future needs

In the UK, there are a number of academic and industry‐led meetings and courses that provide training in the use of HCL. The online Glooko Academy (available in the UK and Ireland) has recently developed CPD‐approved modules for HCL that can be used by all HCPs to increase their knowledge and awareness.^26^ However, confidence can only really be gained by practical experience. This is reflected in results from our survey, which demonstrate that access to specific systems within the clinical environment and practical experience using these systems in relevant patient cohorts were associated with significantly higher confidence ratings. This reinforces the importance of hands‐on experience and highlights ongoing issues surrounding inequitable supply of technologies even within a publicly funded healthcare setting. With wider access to HCL systems as part of the 5‐year implementation, it is important that services incorporate the training of endocrinology residents and the wider multidisciplinary team into service development and implementation plans.

Whilst confidence and awareness in most assessed domains was self‐reported as high, the majority of respondents wanted further training on HCL systems. This need is likely a reflector of the expanding pool of available HCL systems and the diversifying community of pwT1D utilising such technologies. Free text responses suggested a role for national guidelines and patient literature to enable uniformity in clinical practice. While no current evidence directly links self‐reported confidence or awareness among HCPs to safety risks or clinical complications associated with HCL therapy, the well‐established relationship between high‐quality training and optimal outcomes from HCL^25^, ^38^ suggests that further research is warranted. It would be of interest to explore whether variations in self‐reported confidence and awareness, that may reflect training deficits, influence clinical outcomes in those utilising HCL systems.

Equally important is recognition that HCPs inadvertently act as gatekeepers of access to potentially life‐changing therapies. Access to diabetes technologies remains inequitable among underserved groups, even in publicly funded systems^39^, ^40^ and is reflected by findings from our survey, where most respondents reported some or considerable inequities in the provision of HCL systems in their current service. Factors including ethnicity, social background, and differential access to technology continue to influence diabetes outcomes^41^, ^42^, ^43^. However, real‐world evidence demonstrates that when adults with T1D are provided access to technologies, improvements to glycaemia occur irrespective of socioeconomic or ethnic background.^39^, ^44^, ^45^, ^46^ Observations make it difficult to refute a role for inequities in access as a contributor to the poorer diabetes outcomes observed. It is of note that those from the most socio‐economically deprived backgrounds report reduced discussions with HCPs regarding available technologies when compared with the least deprived.^47^ Understanding the role of HCP bias as a contributor to inequities in HCL access is an area that further training can also address.

To further bridge the gap in access to advanced diabetes technologies, multi‐lingual education documents and resources for those with additional hearing, visual, literacy, or numerical processing needs may increase accessibility. Limited digital and health literacy have also been suggested as factors that could affect technology use in certain groups.^48^, ^49^ Consideration of the greater support and time needed for these individuals and the adequate provision of resources to support them and the HCPs delivering their care is key. For some with T1D, remotely accessible training platforms may assist with adapting to and making best use of newer devices.^24^ For example, the successful online DAFNE Closed Loop Essentials course for pwT1D has been accessed by over 5000 patients onboarding to HCL to date.^30^ It is appreciated that platforms may need to be adapted to cater to those with language barriers or sensory needs.

In the UK where healthcare is publicly funded, individuals meeting criteria for HCL funding are typically not required to pay out of pocket. Nevertheless, practical barriers remain, including the need for system‐compatible smartphones and reliable internet access that are not universally available. The NHS and industry are working to overcome such barriers; the NHS is providing smartphones for those who cannot afford them, and some HCL systems have inbuilt internet access.

### 4.5 Strengths and limitations

This is the first study to identify training needs among multidisciplinary HCPs involved in T1D care within a healthcare system where HCL systems are widely endorsed by healthcare policy and increasingly represent standard practice. The survey's breadth, encompassing responses from 135 NHS Trusts, Health Boards and Social care Trusts within the UK, provides robust representation of HCP experiences across various healthcare settings. Both smaller and larger services are represented in this study, and hence we feel that the perspectives of the respondents are generalisable to the wider community of HCPs working in T1D. No single centre dominated the sample, with the highest number of respondents from any one centre being 16, representing approximately 2.5% of the total cohort. This distribution reduces the risk of bias due to overrepresentation from individual sites. However, the low response rate from services outside England may limit the generalisability of findings across all UK regions. Additionally, 28.5% of respondents did not disclose their region of work and further limits the ability to explore geographic disparities in access or confidence. Nevertheless, the recent funding considerations, national implementation programme and therefore wider uptake for HCL systems in T1D care are more focused in England, which may explain the higher response rate in this region (references for NICE TA and NHSE implementation).

The survey was intended for HCPs involved in T1D care. It was distributed directly via professional channels to members of ABCD‐DTN, ABCD as well as other professional organisations (see *Methods: Survey Distribution*). This targeted approach ensured wide inclusion of HCPs involved in T1D care in the UK. However, the total number of HCPs reached through broader dissemination on professional platforms or internal communications within teams is unknown. Consequently, a formal HCP response rate cannot be accurately calculated. It is therefore not possible to assess response bias, which is a limitation of the study design.

Additionally, despite free‐text responses, the primarily quantitative nature of the survey limits deeper qualitative exploration of HCP experiences. Future qualitative research via semi‐structured interviews would further illuminate the nuances of our findings. Given the methodology, we did not assess specific competencies around HCL therapy.

## 5 CONCLUSIONS

This survey demonstrates high overall confidence and awareness among HCPs within the UK regarding commercial HCL systems, reflecting the effectiveness of current educational initiatives. However, targeted training deficits, particularly regarding pregnancy‐specific systems and inequities in access, must be addressed to maximise the potential benefits of these technologies. Enhanced hands‐on exposure and comprehensive, accessible educational resources will be vital to ensuring equitable, safe, and effective use of HCL systems in diverse clinical settings.

## AUTHOR CONTRIBUTIONS

The survey was designed with input and feedback from co‐authors by A.A. and S.H. Data analysis was conducted by A.A., A.L.L., and S.H. A.A. wrote the first draft of the manuscript. All authors critically reviewed and approved the final version of the manuscript. SH is the guarantor of the work and, as such, had full access to all the data in the study and takes responsibility for the integrity of the data and the accuracy of the data analysis.

## FUNDING INFORMATION

S.H. is a recipient of the Medical Research Council Clinical Academic Partnership award (MR/W030004/1). A.A. is supported by an unrestricted educational grant from Abbott Diabetes Care, awarded to S.H. The funders had no role in the design, conduct, analysis, or reporting of the study findings.

## CONFLICT OF INTEREST STATEMENT

A.A. has no conflicts of interest to declare. A.L.L. has received speaker fees and/or support to attend conferences from Dexcom and Novo Nordisk and research support from the Association of British Clinical Diabetologists. K.D. author has received honoraria for travel, advisory boards, and speaker fees from AstraZeneca, Boehringer Ingelheim, Novo Nordisk, Eli Lily, and Sanofi Diabetes. G.G. has received speaker fees and consultation fees from Medtronic, Dexcom, Insulet, Roche, and Sanofi. J.E. has received personal fees from Abbott, Boehringer, Dexcom, Eli Lilly, Glooko, Insulet, Novo Nordisk, Roche, Sanofi, and Ypsomed and research support from Dexcom. P.C. has received personal fees from Abbott, Dexcom, Medtronic, Insulet, Roche, Novo Nordisk, Sanofi, Lilly, Vertex, and CML and research support from Abbott, Medtronic, Dexcom, Insulet, Novo Nordisk. AL has received personal fees from Abbott Diabetes Care, Dexcom, Insulet, Lilly Diabetes, Medtronic, Menarini, Novo Nordisk and Sanofi, and rsearch support from Abbott Diabetes Care and Novo Nordisk. E.G.W. has received personal fees from Abbott, AstraZeneca, Dexcom, Eli Lilly, Embecta, Insulet, Medtronic, Novo Nordisk, Roche, Sanofi, Sinocare, and Ypsomed and research support from Abbott, Embecta, Insulet, Novo Nordisk, and Sanofi. S.H. has served on the advisory board for Tandem, Dexcom, Medtronic; undertaken non‐promotional educational and/ or consultancy work for Abbott UK, Insulet, Dexcom, and Roche; received an unrestricted educational research grant from Abbott UK and an investigator‐initiated research grant from Insulet. ABCD and ABCD‐DTN‐UK receive industry support to support its activities (https://abcd.care/corporate-supporters). The funders had no role in the design, conduct, analysis, or reporting of the study findings.

## PEER REVIEW

The peer review history for this article is available at https://www.webofscience.com/api/gateway/wos/peer-review/10.1111/dom.70027.

## Supporting information


**Data S1.** Supporting information.

## Data Availability

Data available on request from the authors.
