# Supplementary material for: Hybrid closed‐loop systems in UK type 1 diabetes care: National survey of healthcare professional awareness, confidence, and training needs
Source: Diabetes Obes Metab. 2025 Aug 22;27(11):6353–66. doi: 10.1111/dom.70027 (PMC12515757; doi:10.1111/dom.70027)
Supplement: Supplementary file 1 — Data S1. Supporting information. [file DOM-27-6353-s001.docx]

**Supplemental figure 1, total respondents;** 642 respondents participated in the survey. Responses from five participants were excluded from analysis leaving a total cohort of 637 individuals from 135 healthcare organisations within the UK. HCP, healthcare professionals.

**Supplemental figure 2, outpatient encounters,** (1A) number of outpatient clinics per week reported by respondents, (1B) number of people with type 1 diabetes (pwT1D) using hybrid closed loop (HCL) systems encountered in the outpatient setting per week.

**Number of clinics**

**Number of clinics**

**Supplemental table 1 Self disclosed places of work, reported as NHS Trusts, Health boards, Social care trusts or clinics**

| **KEY** | |
| --- | --- |
|  | General Practice within England |
|  | Private Practice in England |
|  | Northern Ireland Social care trust |
|  | Scottish NHS Board |
|  | Welsh NHS Board |
|  | English NHS Trust |

| **Unique Healthcare practices/boards/NHS Trust** | | | |
| --- | --- | --- | --- |
|  |  | Frequency | Percent |
| 1 | Airedale NHS Foundation Trust | 1 | 0.2 |
| 2 | Ashford and St Peter’s Hospitals NHS Foundation Trust | 2 | 0.4 |
| 3 | Barking, Havering and Redbridge University Hospitals NHS Trust | 1 | 0.2 |
| 4 | Barnsley Hospital NHS Foundation Trust | 1 | 0.2 |
| 5 | Bart's Health NHS Trust | 13 | 2.9 |
| 6 | Bedfordshire Hospitals NHS Foundation Trust | 1 | 0.2 |
| 7 | Belfast Health and Social Care Trust | 2 | 0.4 |
| 8 | Berkshire Healthcare NHS Foundation Trust | 3 | 0.7 |
| 9 | Betsi Cadwaladr University Health Board | 2 | 0.4 |
| 10 | Birmingham Women's and Children's NHS Foundation Trust | 1 | 0.2 |
| 11 | Blackpool Teaching Hospitals NHS Foundation Trust | 1 | 0.2 |
| 12 | Blackpool Teaching Hospitals NHS Foundation Trust | 2 | 0.4 |
| 13 | Bolton NHS Foundation Trust | 2 | 0.4 |
| 14 | Bradford Teaching Hospitals NHS Foundation Trust | 3 | 0.7 |
| 15 | Buckinghamshire Healthcare NHS Trust | 1 | 0.2 |
| 16 | Calderdale and Huddersfield NHS Trust | 1 | 0.2 |
| 17 | Cambridge University Hospitals NHS Foundation Trust | 16 | 3.5 |
| 18 | Cardiff and Vale University Health Board | 2 | 0.4 |
| 19 | Chelsea and Westminster Hospital NHS Foundation Trust | 2 | 0.4 |
| 20 | Chesterfield Royal Hospital NHS Foundation Trust | 5 | 1.1 |
| 21 | Countess of Chester Hospital NHS Foundation Trust | 3 | 0.7 |
| 22 | County Durham and Darlington NHS Foundation Trust | 2 | 0.4 |
| 23 | Croydon Health Services NHS Trust | 3 | 0.7 |
| 24 | Cwm Taf Morgannwg University Health Board | 1 | 0.2 |
| 25 | Dartford and Gravesham NHS Trust | 3 | 0.7 |
| 26 | Doncaster & Bassetlaw Teaching Hospitals NHS Trust | 2 | 0.4 |
| 27 | East and North Hertfordshire NHS Trust | 6 | 1.3 |
| 28 | East Cheshire NHS Trust | 1 | 0.2 |
| 29 | East Kent Hospitals University NHS Foundation Trust | 5 | 1.1 |
| 30 | East Lancashire Hospitals NHS Trust | 10 | 2.2 |
| 31 | East Suffolk and North Essex NHS Foundation Trust | 4 | 0.9 |
| 32 | East Sussex Healthcare NHS Trust | 1 | 0.2 |
| 33 | Frimley Health NHS Foundation Trust | 2 | 0.4 |
| 34 | Gateshead Health NHS Foundation Trust | 2 | 0.4 |
| 35 | General practice 1 | 1 | 0.2 |
| 36 | General practice 2 | 1 | 0.2 |
| 37 | Gloucestershire Hospitals NHS Foundation Trust | 3 | 0.7 |
| 38 | Great Ormond Street Hospital for Children NHS Foundation Trust | 1 | 0.2 |
| 39 | Guys and St Thomas' NHS Foundation Trust | 7 | 1.5 |
| 40 | Hampshire Hospitals NHS Foundation Trust | 3 | 0.7 |
| 41 | Harrogate District Foundation Trust | 3 | 0.7 |
| 42 | Homerton Healthcare NHS Foundation Trust | 1 | 0.2 |
| 43 | Hull University Teaching Hospitals NHS Trust | 2 | 0.4 |
| 44 | Hywel Dda University Health Board | 1 | 0.2 |
| 45 | Imperial College Healthcare NHS Trust | 3 | 0.7 |
| 46 | Isle of Wight NHS Trust | 2 | 0.4 |
| 47 | James Paget University Hospital NHS Foundation Trust | 1 | 0.2 |
| 48 | Kettering General Hospital NHS Foundation Trust | 1 | 0.2 |
| 49 | King's College Hospital NHS Foundation Trust: | 8 | 1.8 |
| 50 | Kingston Hospital NHS Trust | 2 | 0.4 |
| 51 | Lancashire Teaching Hospitals NHS Foundation Trust | 2 | 0.4 |
| 52 | Leeds Teaching Hospitals NHS Trust | 10 | 2.2 |
| 53 | Lewisham and Greenwich NHS Trust | 6 | 1.3 |
| 54 | London North West University Healthcare NHS Trust | 4 | 0.9 |
| 55 | Maidstone and Tunbridge Wells NHS Trust | 2 | 0.4 |
| 56 | Manchester University NHS Foundation Trust | 11 | 2.4 |
| 57 | Medway NHS Foundation Trust | 1 | 0.2 |
| 58 | Mersey and West Lancashire Teaching Hospitals NHS Trust | 10 | 2.2 |
| 59 | Mid and South Essex NHS Foundation Trust | 7 | 1.5 |
| 60 | Mid Yorkshire Teaching NHS Trust | 2 | 0.4 |
| 61 | Milton Keynes University Hospital NHS Foundation Trust | 1 | 0.2 |
| 62 | Newcastle upon Tyne Hospitals NHS Foundation Trust | 5 | 1.1 |
| 63 | NHS Ayrshire and Arran | 1 | 0.2 |
| 64 | NHS Fife | 2 | 0.4 |
| 65 | NHS Forth Valley | 1 | 0.2 |
| 66 | NHS Grampian | 1 | 0.2 |
| 67 | NHS Greater Glasgow and Clyde | 12 | 2.6 |
| 68 | NHS Lanarkshire | 1 | 0.2 |
| 69 | NHS Lothian | 8 | 1.8 |
| 70 | NHS Wales | 2 | 0.4 |
| 71 | Norfolk and Norwich University Hospitals NHS Foundation Trust | 5 | 1.1 |
| 72 | North Cumbria Integrated Care NHS Foundation Trust | 1 | 0.2 |
| 73 | North Middlesex University Hospital NHS Trust | 3 | 0.7 |
| 74 | North West Anglia NHS Foundation Trust | 5 | 1.1 |
| 75 | Northern Care Alliance NHS Group | 10 | 2.2 |
| 76 | Northern Lincolnshire and Goole NHS Foundation Trust | 2 | 0.4 |
| 77 | Northumbria Healthcare NHS Foundation Trust | 2 | 0.4 |
| 78 | Nottingham University Hospitals NHS Trust | 3 | 0.7 |
| 79 | Oxford University Hospitals NHS Foundation Trust | 5 | 1.1 |
| 80 | Portsmouth Hospitals University NHS Trust | 1 | 0.2 |
| 81 | Princess Alexandra Hospital NHS Trust | 2 | 0.4 |
| 82 | Private practice 1 | 1 | 0.2 |
| 83 | Private practice 2 | 1 | 0.2 |
| 84 | Royal Berkshire NHS Foundation Trust | 1 | 0.2 |
| 85 | Royal Cornwall Hospitals NHS Trust | 5 | 1.1 |
| 86 | Royal Devon University Healthcare NHS Foundation Trust | 8 | 1.8 |
| 87 | Royal Free London NHS Foundation Trust | 5 | 1.1 |
| 88 | Royal Surrey NHS Foundation trust | 4 | 0.9 |
| 89 | Royal United Hospitals NHS Foundation Trust | 1 | 0.2 |
| 90 | Royal Wolverhampton Hospitals NHS Trust | 2 | 0.4 |
| 91 | Sandwell and West Birmingham Hospitals NHS Trust | 1 | 0.2 |
| 92 | Sheffield Children's NHS Foundation Trust | 3 | 0.7 |
| 93 | Sheffield Teaching Hospitals NHS Foundation Trust | 5 | 1.1 |
| 94 | Sherwood Forest Hospitals NHS Foundation Trust | 3 | 0.7 |
| 95 | Shrewsbury and Telford Hospitals NHS Trust | 1 | 0.2 |
| 96 | Somerset NHS Foundation Trust | 15 | 3.3 |
| 97 | South Tees Hospitals NHS Foundation Trust | 5 | 1.1 |
| 98 | South Tyneside and Sunderland NHS Foundation Trust | 10 | 2.2 |
| 99 | South Warwickshire University NHS Foundation Trust | 1 | 0.2 |
| 100 | St George's University Hospitals NHS Foundation Trust | 1 | 0.2 |
| 101 | St Helens and Knowsley Teaching Hospitals NHS Trust | 1 | 0.2 |
| 102 | Stockport NHS Foundation Trust | 2 | 0.4 |
| 103 | Surrey and Sussex Healthcare NHS Trust | 2 | 0.4 |
| 104 | Sussex Partnership NHS Foundation Trust | 1 | 0.2 |
| 105 | Swansea Bay University Health Board, | 2 | 0.4 |
| 106 | Tameside and Glossop Integrated Care NHS Foundation Trust | 4 | 0.9 |
| 107 | The Dudley Group NHS Foundation Trust | 2 | 0.4 |
| 108 | The Hillingdon Hospitals NHS Foundation Trust | 1 | 0.2 |
| 109 | The Northern Care Alliance NHS Foundation Trust | 1 | 0.2 |
| 110 | The Queen Elizabeth Hospital Kings Lynn NHS Foundation Trust: | 2 | 0.4 |
| 111 | The Royal United Hospitals Bath NHS Foundation Trust | 8 | 1.8 |
| 112 | The Western Health and Social Care Trust | 2 | 0.4 |
| 113 | Torbay and South Devon NHS Foundation Trust, | 1 | 0.2 |
| 114 | United Lincolnshire Hospitals NHS Trust | 1 | 0.2 |
| 115 | University College London Hospitals NHS Foundation Trust | 6 | 1.3 |
| 116 | University Hospital Southampton NHS Foundation Trust | 1 | 0.2 |
| 117 | University Hospitals Birmingham NHS Foundation Trust | 9 | 2 |
| 118 | University Hospitals Bristol and Weston NHS Foundation Trust | 5 | 1.1 |
| 119 | University Hospitals Coventry and Warwickshire NHS Trust | 1 | 0.2 |
| 120 | University Hospitals Dorset NHS Foundation Trust | 1 | 0.2 |
| 121 | University Hospitals Morecambe Bay NHS Foundation Trust | 2 | 0.4 |
| 122 | University Hospitals of Derby and Burton NHS Foundation Trust | 11 | 2.4 |
| 123 | University Hospitals of Leicester NHS Trust | 5 | 1.1 |
| 124 | University Hospitals of Morecambe Bay NHS Foundation Trust | 4 | 0.9 |
| 125 | University Hospitals of North Midlands NHS Trust | 1 | 0.2 |
| 126 | University Hospitals Plymouth NHS Trust | 7 | 1.5 |
| 127 | University Hospitals Sussex NHS Trust | 6 | 1.3 |
| 128 | Walsall Healthcare NHS Trust | 1 | 0.2 |
| 129 | Warrington and Halton Teaching Hospitals NHS Foundation Trust | 3 | 0.7 |
| 130 | West Hertfordshire Teaching Hospitals NHS Trust | 1 | 0.2 |
| 131 | West Suffolk NHS Foundation Trust | 3 | 0.7 |
| 132 | Wirral University Teaching Hospital NHS Foundation Trust | 2 | 0.4 |
| 133 | Wrightington, Wigan and Leigh Teaching Hospitals NHS Foundation Trust | 4 | 0.9 |
| 134 | Wye Valley NHS Trust | 1 | 0.2 |
| 135 | York and Scarborough Teaching Hospitals NHS Foundation Trust | 3 | 0.7 |
|  | **Total** | **455** | **100** |

**Supplementary table 3;** Spearman’s correlation analyses revealed significant relationships between awareness in all safety considerations

| **Awareness (Likert 1-7)** |  | | **Awareness (Likert 1-7)** | | | | |
| --- | --- | --- | --- | --- | --- | --- | --- |
|  |  |  | **When cannulae failure should be expected** | **Sick day rules and managing unexplained hyperglycaemia** | **Managing hypoglycaemia** | **When to advise a pwT1DM use manual mode** | **When to advise a PwT1DM to come out of closed loop and use their injections** |
|  | **When cannulae failure should be expected** | Correlation Coefficient |  | .856^**^ | .787^**^ | .806^**^ | .819^**^ |
|  |  | Sig. (2-tailed) |  | <.001 | <.001 | <.001 | <.001 |
|  |  | N |  | 634 | 634 | 634 | 630 |
|  | **Sick day rules and managing unexplained hyperglycaemia** | Correlation Coefficient | .856^**^ |  | .868^**^ | .844^**^ | .855^**^ |
|  |  | Sig. (2-tailed) | <.001 |  | <.001 | <.001 | <.001 |
|  |  | N | 634 |  | 635 | 635 | 631 |
|  | **Managing hypoglycaemia** | Correlation Coefficient | .787^**^ | .868^**^ |  | .819^**^ | .815^**^ |
|  |  | Sig. (2-tailed) | <.001 | <.001 |  | <.001 | <.001 |
|  |  | N | 634 | 635 |  | 635 | 632 |
|  | **When to advise a pwT1DM use manual mode** | Correlation Coefficient | .806^**^ | .844^**^ | .819^**^ |  | .924^**^ |
|  |  | Sig. (2-tailed) | <.001 | <.001 | <.001 |  | <.001 |
|  |  | N | 634 | 635 | 635 |  | 631 |
|  | **When to advise a PwT1DM to come out of closed loop and use their injections** | Correlation Coefficient | .819^**^ | .855^**^ | .815^**^ | .924^**^ |  |
|  |  | Sig. (2-tailed) | <.001 | <.001 | <.001 | <.001 |  |
|  |  | N | 630 | 631 | 632 | 631 |  |

**Supplemental table 4;** Awareness of safety considerations when compared between professional role

|  | **Awareness (Likert 1-7)** | | | | |
| --- | --- | --- | --- | --- | --- |
|  | When to suspect cannula failure | Sick day rules and managing unexplained hyperglycaemia | The management of hypoglycaemia | When to advise manual | When to use MDI |
| Endocrinologists | 6.00 [6.00, 7.00] | 6.00 [6.00, 7.00] | 6.00 [6.00, 7.00] | 6.00 [5.00, 7.00] | 6.00 [5.00, 7.00] |
| Diabetes nurses | 7.00 [6.00, 7.00] | 7.00 [6.00, 7.00] | 7.00 [6.00, 7.00] | 6.50 [6.00, 7.00] | 7.00 [6.00, 7.00] |
| p | .011 | .067 | .004 | .003 | .060 |
| Endocrinologists | 6.00 [6.00, 7.00] | 6.00 [6.00, 7.00] | 6.00 [6.00, 7.00] | 6.00 [5.00, 7.00] | 6.00 [5.00, 7.00] |
| Endocrinology residents | 5.00 [3.00, 6.00] | 5.00 [3.00, 6.00] | 5.00 [3.00, 6.00] | 5.00 [3.00, 5.00] | 5.00 [3.00, 6.00] |
| p | <.001 | <.001 | <.001 | <.001 | <.001 |
| Endocrinology residents | 5.00 [3.00, 6.00] | 5.00 [3.00, 6.00] | 5.00 [3.00, 6.00] | 5.00 [3.00, 5.00] | 5.00 [3.00, 6.00] |
| Diabetes nurses | 7.00 [6.00, 7.00] | 7.00 [6.00, 7.00] | 7.00 [6.00, 7.00] | 6.50 [6.00, 7.00] | 7.00 [6.00, 7.00] |
| p | <.001 | <.001 | <.001 | <.001 | <.001 |

**Supplemental files – survey content**

Hybrid Closed Loop (HCL) questionnaire for healthcare professionals

**Top of Form**

**Demographics**

What age range do you work with?

 Adults

 Young adults (18-25)

 Paediatrics (mostly under 19)

 Adults and paediatrics

 Adults and young adults

 Young adults and paediatrics

What is your role?

Consultant in Diabetes and Endocrinology

 Speciality trainee doctor (ST3-7)

 Trainee doctor (not in speciality training)

 General Practitioner

 Doctor not in training

 Diabetes nurse

 Diabetes dietitian

 Psychologist

 Psychiatrist

 Other…

Which Trust or Clinic do you work in?

If you prefer not to answer please write N/A

What is your practice type?

 NHS teaching hospital

 NHS District general hospital

 Community Diabetes service

 Other…

Which age range do you belong to?

 18-25

 26-35

 36-45

 46-55

 56-70

 >70

What is your gender identity?

 Female

 Male

 Transgender

 Non binary

 Other

 Prefer not to say

**HCL - Exposure**

How many diabetes clinics do you take part in on a weekly basis?

 0

 1

 2

 3

 4

 5

 More than 5

Do you work in a trust/clinic where there are specific clinics for people with type 1 diabetes (pwT1DM) using insulin pump therapy (CSII) including those on HCL systems?

 Yes

 No

How many pwT1DM do you encounter in the outpatient setting making use of hybrid closed loop (HCL) systems on an average week?

 0

 1

 2

 3

 4

 5

 6

 More than 6

If you are involved in inpatient diabetes care, have you been involved in the inpatient care of pwT1DM using HCL?

 Yes

 No

 Not applicable

If you are involved in inpatient diabetes care, have you been involved in the inpatient care of pwT1DM undergoing surgery?

 Yes

 No

 Not applicable

Are you involved in the care of of pwT1DM using HCL during pregnancy?

 Yes

 No

 Not applicable

**HCL - starts and patient education**

Are you involved in HCL starts in your trust/clinic?

 Yes

 No

How aware are you of the following:

1 - Completely unaware | 2 - Mostly unaware | 3 - Somewhat unaware | 4 - Neutral / awareness undetermined | 5 - Somewhat aware | 6 - Mostly aware | 7 - Completely aware

| **Questions** | **1** | **2** | **3** | **4** | **5** | **6** | **7** |
| --- | --- | --- | --- | --- | --- | --- | --- |
| The procedure for HCL starts in your trust/clinic | 1 | 2 | 3 | 4 | 5 | 6 | 7 |
| What services there are presently in your trust/clinic to assist and educate pwT1DM regarding HCL? | 1 | 2 | 3 | 4 | 5 | 6 | 7 |
| The education needed for a pwT1DM prior to commencing HCL therapy? | 1 | 2 | 3 | 4 | 5 | 6 | 7 |
| The concept of onboarding when it comes to HCL systems? | 1 | 2 | 3 | 4 | 5 | 6 | 7 |
| The services available for onboarding in your clinic/trust? | 1 | 2 | 3 | 4 | 5 | 6 | 7 |

How confident are you in being able to:

1 - Not at all confident | 2 - Slightly confident | 3 - Somewhat confident | 4 - Moderately confident | 5 - Confident | 6 - Very confident | 7 - Completely confident

| **Questions** | **1** | **2** | **3** | **4** | **5** | **6** | **7** |
| --- | --- | --- | --- | --- | --- | --- | --- |
| Assist a person with type 1 diabetes in making a decision about which HCL system would be right for them? | 1 | 2 | 3 | 4 | 5 | 6 | 7 |
| Signpost pwT1DM considering HCL therapies toward online educational resources and training platforms? | 1 | 2 | 3 | 4 | 5 | 6 | 7 |

**HCL - currently available systems**

How confident are you in advising pwT1DM on the use of the following HCL systems:

1 - Not at all confident | 2 - Slightly confident | 3 - Somewhat confident | 4 - Moderately confident | 5 - Confident | 6 - Very confident | 7 - Completely confident

| **Questions** | **1** | **2** | **3** | **4** | **5** | **6** | **7** |
| --- | --- | --- | --- | --- | --- | --- | --- |
| Control IQ (Tandem T-slim) | 1 | 2 | 3 | 4 | 5 | 6 | 7 |
| SmartGuard (Medtronic 780G) | 1 | 2 | 3 | 4 | 5 | 6 | 7 |
| Smart Adjust (Omnipod 5) | 1 | 2 | 3 | 4 | 5 | 6 | 7 |
| CamAPS FX (Ypsopump) | 1 | 2 | 3 | 4 | 5 | 6 | 7 |
| CamAPS Fx (Dana-i) | 1 | 2 | 3 | 4 | 5 | 6 | 7 |

For the current commercially available HCL systems in the UK, how aware are you of:

1 - Completely unaware | 2 - Mostly unaware | 3 - Somewhat unaware | 4 - Neutral / awareness undetermined | 5 - Somewhat aware | 6 - Mostly aware | 7 - Completely aware

| **Questions** | **1** | **2** | **3** | **4** | **5** | **6** | **7** |
| --- | --- | --- | --- | --- | --- | --- | --- |
| How each system determines insulin delivery? | 1 | 2 | 3 | 4 | 5 | 6 | 7 |
| What glucose target ranges can be specified? | 1 | 2 | 3 | 4 | 5 | 6 | 7 |
| How the system adjusts insulin and offers corrections? | 1 | 2 | 3 | 4 | 5 | 6 | 7 |
| What settings can be adjusted and their impact on glycaemia? | 1 | 2 | 3 | 4 | 5 | 6 | 7 |
| Reasons for a system-initiated exit from closed loop? | 1 | 2 | 3 | 4 | 5 | 6 | 7 |

Can your trust/clinic offer the following HCL systems?

| **Questions** | **Yes** | **No** | **Unsure** |
| --- | --- | --- | --- |
| Control IQ (Tandem T-slim) | Yes | No | Unsure |
| SmartGuard (Medtronic 780G) | Yes | No | Unsure |
| Smart Adjust (Omnipod 5) | Yes | No | Unsure |
| CamAPS FX (Ypsopump) | Yes | No | Unsure |
| CamAPS Fx (Dana-i) | Yes | No | Unsure |

**HCL – data interpretation and technical issues**

How confident are you in reviewing HCL data?

1 Not at all confident | 2 Slightly confident | 3 Somewhat confident | 4 Moderately confident | 5 Confident | 6 Very confident | 7 Completely confident

How confident are you of which parameters are adjustable in the following systems?

1 - Not at all confident | 2 - Slightly confident | 3 - Somewhat confident | 4 - Moderately confident | 5 - Confident | 6 - Very confident | 7 - Completely confident

| **Questions** | **1** | **2** | **3** | **4** | **5** | **6** | **7** |
| --- | --- | --- | --- | --- | --- | --- | --- |
| Control IQ ( Tandem) | 1 | 2 | 3 | 4 | 5 | 6 | 7 |
| SmartGuard (Medtronic) | 1 | 2 | 3 | 4 | 5 | 6 | 7 |
| SmartAdjust (Omnipod) | 1 | 2 | 3 | 4 | 5 | 6 | 7 |
| Cam APS (Ypsomed/Dana) | 1 | 2 | 3 | 4 | 5 | 6 | 7 |

How confident are you in making adjustments to these parameters in each system?

1 - Not at all confident | 2 - Slightly confident | 3 - Somewhat confident | 4 - Moderately confident | 5 - Confident | 6 - Very confident | 7 - Completely confident

| **Questions** | **1** | **2** | **3** | **4** | **5** | **6** | **7** |
| --- | --- | --- | --- | --- | --- | --- | --- |
| Control IQ ( Tandem) | 1 | 2 | 3 | 4 | 5 | 6 | 7 |
| SmartGuard (Medtronic) | 1 | 2 | 3 | 4 | 5 | 6 | 7 |
| SmartAdjust (Omnipod) | 1 | 2 | 3 | 4 | 5 | 6 | 7 |
| Cam APS (Ypsomed/Dana) | 1 | 2 | 3 | 4 | 5 | 6 | 7 |

How confident are you in identifying and managing the following technical issues related to currently available commercial HCL therapies

1 - Not at all confident | 2 - Slightly confident | 3 - Somewhat confident | 4 - Moderately confident | 5 - Confident | 6 - Very confident | 7 - Completely confident

| **Questions** | **1** | **2** | **3** | **4** | **5** | **6** | **7** |
| --- | --- | --- | --- | --- | --- | --- | --- |
| Set failures | 1 | 2 | 3 | 4 | 5 | 6 | 7 |
| Pump failures | 1 | 2 | 3 | 4 | 5 | 6 | 7 |
| Connectivity issues | 1 | 2 | 3 | 4 | 5 | 6 | 7 |

How confident do you feel in being able to upload data from current commercially available HCL systems?

1 - Not at all confident | 2 - Slightly confident | 3 - Somewhat confident | 4 - Moderately confident | 5 - Confident | 6 - Very confident | 7 - Completely confident

| **Questions** | **1** | **2** | **3** | **4** | **5** | **6** | **7** |
| --- | --- | --- | --- | --- | --- | --- | --- |
| Control IQ (Tandem) | 1 | 2 | 3 | 4 | 5 | 6 | 7 |
| SmartGuard (Medtronic) | 1 | 2 | 3 | 4 | 5 | 6 | 7 |
| SmartAdjust (Omnipod) | 1 | 2 | 3 | 4 | 5 | 6 | 7 |
| Cam APS (Ypsomed/Dana) | 1 | 2 | 3 | 4 | 5 | 6 | 7 |

**HCL – safety considerations**

What safety strategies are you aware of to avoid problematic hyperglycaemia and DKA in those using HCL?

How aware are you of:

1 - Completely unaware | 2 - Mostly unaware | 3 - Somewhat unaware | 4 - Neutral / awareness undetermined | 5 - Somewhat aware | 6 - Mostly aware | 7 - Completely aware

| **Questions** | **1** | **2** | **3** | **4** | **5** | **6** | **7** |
| --- | --- | --- | --- | --- | --- | --- | --- |
| The circumstances when cannula failure should be suspected? | 1 | 2 | 3 | 4 | 5 | 6 | 7 |
| Sick day rules and managing unexplained hyperglycaemia in pwT1DM using HCL? | 1 | 2 | 3 | 4 | 5 | 6 | 7 |
| The management of hypoglycaemia in those using HCL? | 1 | 2 | 3 | 4 | 5 | 6 | 7 |
| When to advise a pwT1DM to come out of closed loop and use their system in manual (open-loop) mode? | 1 | 2 | 3 | 4 | 5 | 6 | 7 |
| When to advise a PwT1DM to come out of closed loop and use their injections? | 1 | 2 | 3 | 4 | 5 | 6 | 7 |
|  |  |  |  |  |  |  |  |

**HCL- special considerations**

Are you aware of which of the current commercially available HCL systems are licenced for use in pregnancy?

Yes/ No/  Somewhat

How confident are you managing/ advising on the management of:

1 - Not at all confident | 2 - Slightly confident | 3 - Somewhat confident | 4 - Moderately confident | 5 - Confident | 6 - Very confident | 7 - Completely confident

| **Questions** | **1** | **2** | **3** | **4** | **5** | **6** | **7** |
| --- | --- | --- | --- | --- | --- | --- | --- |
| Glycaemia during exercise for pwT1DM using HCL? | 1 | 2 | 3 | 4 | 5 | 6 | 7 |
| Glycaemia in pregnancy for pwT1DM using HCL? | 1 | 2 | 3 | 4 | 5 | 6 | 7 |
| The use of HCL therapy in a pwT1DM currently an inpatient? | 1 | 2 | 3 | 4 | 5 | 6 | 7 |
| HCL therapy in a pwT1DM who is due to undergo surgery? | 1 | 2 | 3 | 4 | 5 | 6 | 7 |

**HCL- current training and future needs**

Would you like further training on HCL therapies?

Yes/No /Somewhat

How would you like training for HCL to be delivered?

Please rank from most to least preferred (top to bottom) by dragging the available options

|  |
| --- |
| Remote learning (modules accessible in your own time) |
| Remote learning (live streamed lectures) |
| Face to face locally delivered training |
| Regional/national training face to face |

**HCL- unmet needs**

To what extent do you feel there are inequalities in the provision of HCL therapies in your service?

1 No inequalities | 2 Some inequalities | 3 Considerable inequalities | 4 Significant inequalities | 5 Extreme inequalities

On a scale of 1-7, how effective do you feel the following strategies would be to address the inequalities in provision of HCL therapies?

1 - Not effective at all | 2 - Slightly effective | 3 - Somewhat effective | 4 - Moderately effective | 5 - Effective | 6 - Very effective | 7 - Extremely effective

| **Questions** | **1** | **2** | **3** | **4** | **5** | **6** | **7** |
| --- | --- | --- | --- | --- | --- | --- | --- |
| Tailored education for those with lower digital literacy | 1 | 2 | 3 | 4 | 5 | 6 | 7 |
| Easy to read written materials | 1 | 2 | 3 | 4 | 5 | 6 | 7 |
| Consultations assisted by translation and interpretation services for those with limited or no ability to speak English | 1 | 2 | 3 | 4 | 5 | 6 | 7 |
| Educational resources and other patient-facing materials provided in common non-English languages | 1 | 2 | 3 | 4 | 5 | 6 | 7 |
| Alterations in product design for those with visual impairment | 1 | 2 | 3 | 4 | 5 | 6 | 7 |
| Alterations in product design for those with dexterity issues | 1 | 2 | 3 | 4 | 5 | 6 | 7 |

We are interested in understanding issues that will impact implementation of HCL systems in your services. Can you highlight any issues your service may encounter that will affect its ability to provide HCL technologies on a larger scale (e.g. issues with implementing NICE TA for HCL if in England or Wales)?

Any other comments or queries

E-mail address (If willing to share)

Bottom of Form
